# The Evolution of Coral Reef under Changing Climate: A Scientometric Review

**DOI:** 10.3390/ani13050949

**Published:** 2023-03-06

**Authors:** Chandra Segaran Thirukanthan, Mohamad Nor Azra, Fathurrahman Lananan, Gianluca Sara’, Inga Grinfelde, Vite Rudovica, Zane Vincevica-Gaile, Juris Burlakovs

**Affiliations:** 1 Institute of Marine Biotechnology (IMB), Universiti Malaysia Terengganu (UMT), Kuala Nerus 21030, Terengganu, Malaysia; 2 Research Center for Marine and Land Bioindustry, Earth Sciences and Maritime Organization, National Research and Innovation Agency (BRIN), Pemenang 83352, Indonesia; 3 East Coast Environmental Research Institute, Universiti Sultan Zainal Abidin (UniSZA), Gong Badak Campus, Kuala Nerus 21300, Terengganu, Malaysia; 4 Laboratory of Ecology, Earth and Marine Sciences Department, University of Palermo, 90133 Palermo, Italy; 5 Laboratory of Forest and Water Resources, Latvia University of Life Sciences and Technologies, LV-3001 Jelgava, Latvia; 6 Department of Analytical Chemistry, University of Latvia, LV-1004 Riga, Latvia; 7 Department of Environmental Science, University of Latvia, LV-1004 Riga, Latvia; 8 Mineral and Energy Economy Research Institute of the Polish Academy of Sciences, 31-261 Krakow, Poland

**Keywords:** coral bleaching, coral disease, coral growth, elevated carbon dioxide, phase shift, ocean acidification, sea level rise, Symbiodinium, surface temperature, future scenario

## Abstract

**Simple Summary:**

Coral reefs are vital ecosystems with high biodiversity and ecological services for coastal communities. Climate change is accelerating, with detrimental consequences on coral reefs and related communities, but it is challenging to keep up with the literature given its current rapid expansion. The current review foresees three future trends in the area of coral reefs and climate change, including (i) incorporating future scenarios, (ii) climate-induced temperature changes, and (iii) adaptation strategies, which are expected to move society closer to the following Sustainable Development Goal: 13 Climate Action.

**Abstract:**

In this scientometric review, we employ the Web of Science Core Collection to assess current publications and research trends regarding coral reefs in relation to climate change. Thirty-seven keywords for climate change and seven keywords for coral reefs were used in the analysis of 7743 articles on coral reefs and climate change. The field entered an accelerated uptrend phase in 2016, and it is anticipated that this phase will last for the next 5 to 10 years of research publication and citation. The United States and Australia have produced the greatest number of publications in this field. A cluster (i.e., focused issue) analysis showed that coral bleaching dominated the literature from 2000 to 2010, ocean acidification from 2010 to 2020, and sea-level rise, as well as the central Red Sea (Africa/Asia), in 2021. Three different types of keywords appear in the analysis based on which are the (i) most recent (2021), (ii) most influential (highly cited), and (iii) mostly used (frequently used keywords in the article) in the field. The Great Barrier Reef, which is found in the waters of Australia, is thought to be the subject of current coral reef and climate change research. Interestingly, climate-induced temperature changes in “ocean warming” and “sea surface temperature” are the most recent significant and dominant keywords in the coral reef and climate change area.

## 1. Introduction

Scleractinians, or stony corals, emerged during the Cambrian period and constructed the earliest reefs, dating to approximately 410 million years ago [1,2,3]. Five major coral extinctions have occurred since then, all of which have been linked to rising temperatures and higher levels of carbon dioxide in the atmosphere [2,4].

While coral reef research commenced more than 100 years ago, concern over the state of coral reefs is relatively recent, occurring only in the last four decades. In 1981, at the 4th International Coral Reef Symposium, Edgardo Gomez initiated the conversation on threats to coral reefs by presenting his concerns to the scientific community [5,6]. Coral reefs are considered an important marine resource for coastal communities, and the conference participants were mainly focused on coral reef management and environmental impacts and related fisheries activities [5,7,8]. In addition to the natural stresses that have always existed on coral reefs, such as storms, freshwater inundation, and seismic and volcanic events, there is growing evidence of new emerging threats potentially causing global damage to coral reefs [7,8,9,10].

At current extinction rates, it is estimated that we are commencing the sixth mass extinction event [11,12,13], with individual extinctions occurring approximately 1000-fold faster than the expected background extinction rate. Theoretically, species extinctions occur at a rate proportional to the rate of speciation or the creation of new species [14]. Current extinction rates are much higher than speciation rates. This is largely due to the fact of anthropogenic factors [15,16], such as habitat destruction [17], deforestation [18], pollution [7], ocean acidification, climate change resulting from greenhouse gas emissions, and overexploitation of ecological resources [19,20,21]. It is estimated that 75% of species will go extinct unless human pressures on the environment are scaled back soon [20,22,23,24,25]. Further management efforts are required to reduce the impacts of climate change and human anthropogenic stress towards coral reef communities [7]. 

**Figure 1:**
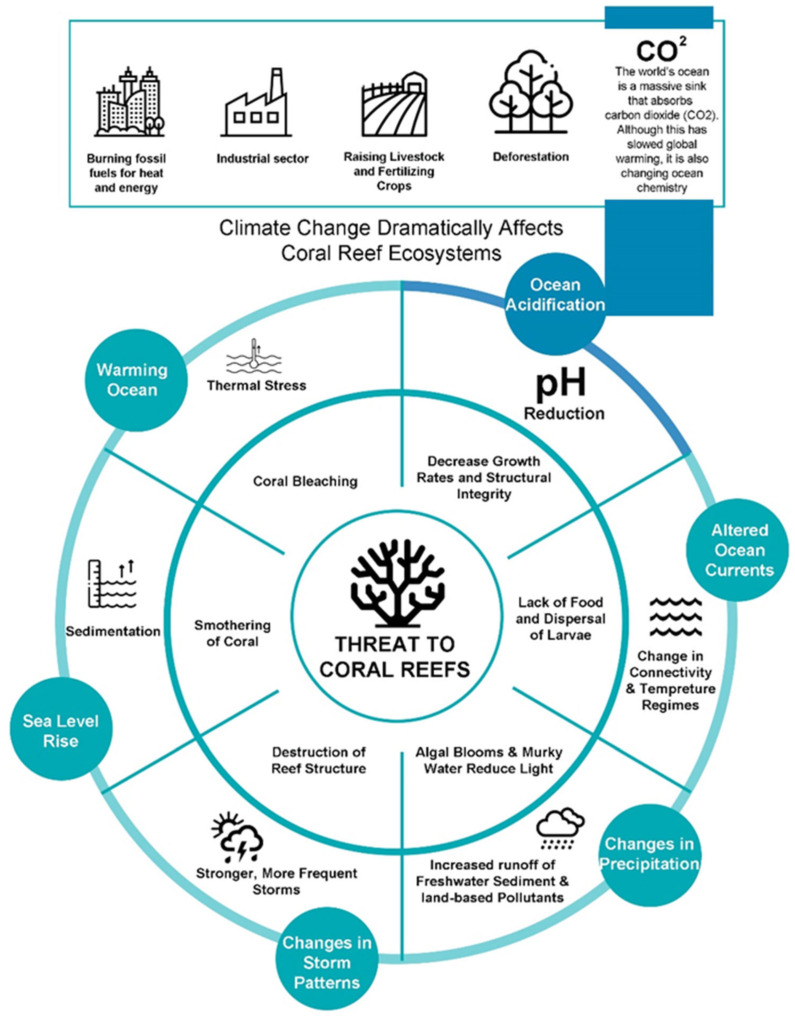
Threats to coral reefs posed by factors related to climate change.

Anthropogenic pressures on reefs have been the dominant factor damaging coral reefs through a range of stresses (Figure 1). Unsustainable land-based human activities, such as deforestation, poorly regulated agriculture, and urban/industrial development, are major contributors to the release of excessive sediments and nutrients into the environment [26,27,28]. Increases in human-caused greenhouse gas emissions are the primary factor in the current climatic shift around the world [29,30]. The ocean acts as a massive sink that absorbs carbon dioxide, resulting in the acidification of the oceans [31,32]. Coral bleaching and widespread damage to the coral reef ecosystem have become increasingly common because of thermal stress brought on by rising ocean temperatures [33]. Lack of food to sustain the coral reef ecosystem and disruption of larvae dispersal are both attributable to altered currents, upwelling, and/or vertical mixing brought on by changing currents and winds [34]. Storms and cyclones are “agents of mortality” on coral reefs and can have a direct impact on the structure and local distribution of coral reef assemblages, especially through the large waves they produce [35]. Reduced salinity caused by heavy rainfall and enhanced surface run-off onto nearshore reefs during cyclones, storms, and heavy precipitation rates can lead to algal blooms and other devastating results [36,37]. The rise in sea level, caused by thermal expansion and the melting of ice on land, has varied across different regions of the world over the past century, with an average increase of approximately 20 cm [38]. The sedimentary mechanisms triggered by the rising sea levels have the potential to intensify and jeopardize crucial physiological reef processes, such as photosynthesis, feeding, and recruitment, thereby posing a severe threat to coral reefs and related ecosystems, such as seagrass meadows and mangrove forests [39,40]. This threat, coupled with increasing carbon dioxide (CO_2_) emissions, can negatively impact these vital ecosystems. Without effective local measures and a concerted effort to reduce carbon dioxide emissions, these effects are projected to intensify, leading to an unprecedented degradation of marine biodiversity and ecological balance [41,42].

Given the length of time that scientists have been studying climate change [43], the sheer volume of published research in the field can make it difficult for scientists to develop an overview of the topic [44]. Bibliometric analysis can be used to provide an overview of voluminous scientific literature [45]. Research output in each field can be charted in terms of its characteristics and evolution through quantitative examination of publication data [45,46]. Performance and research patterns of authors, journals, countries, and institutions can all be evaluated with the help of bibliometric methods, and patterns of collaboration between these entities can be identified and quantified [47]. A research domain’s multidisciplinary nature and the variety of journals publishing on a given topic can be inferred from the subject categories assigned to publications and the number of journals publishing on a given topic. The most recent developments, research directions, and top-of-mind issues in a particular field can be gleaned from bibliometrics [48]. In addition, bibliometrics can be a useful tool for guiding scientific policy. Findings from bibliometric analyses not only inform researchers and policymakers but also aid in the distribution of funds for scientific investigation [49].

## 2. Scientometric Analysis

Software programs, such as VOSviewer (Centre for Science and Technology Studies (CWTS), Leiden University, Leiden, The Netherlands), Pajek (University of Ljubljana, Ljubljana, Slovenia), and CiteSpace (Drexel University, Philadelphia, PA, USA) can be used to create scientometric visualizations. CiteSpace is a scientometric-based analysis tool that provides two main outputs for researchers. Firstly, it includes three central concepts: burst detection, betweenness centrality, and heterogeneous networks. Secondly, CiteSpace addresses three practical issues: identifying the nature of a research front, labeling specialties, and detecting emerging trends and abrupt changes in a timely manner. Identifying these outputs involves six main procedures: time slicing, thresholding, modelling, pruning, merging, and mapping. CiteSpace’s functionalities, such as dual map overlay, burst detection, cluster explorer, and timeline view, are particularly helpful in identifying the current research trends of a specific field. Researchers can use this information to gain insights into the overall state of research in a given field, the pace of advancement, and the most prominent areas of interest [50,51].

Briefly, the dual map overlay graphically identifies both original documents and cocited networks and expresses their relationships by connecting them with lines. Burst detection refers to a frequency surge of a specific knowledge domain. A cluster is defined as the frequency of citations of cited references. A timeline arranges papers in chronological rows, with each cluster represented by one row and papers represented as nodes [52].

## 3. Objective of the Review

**Figure 2:**
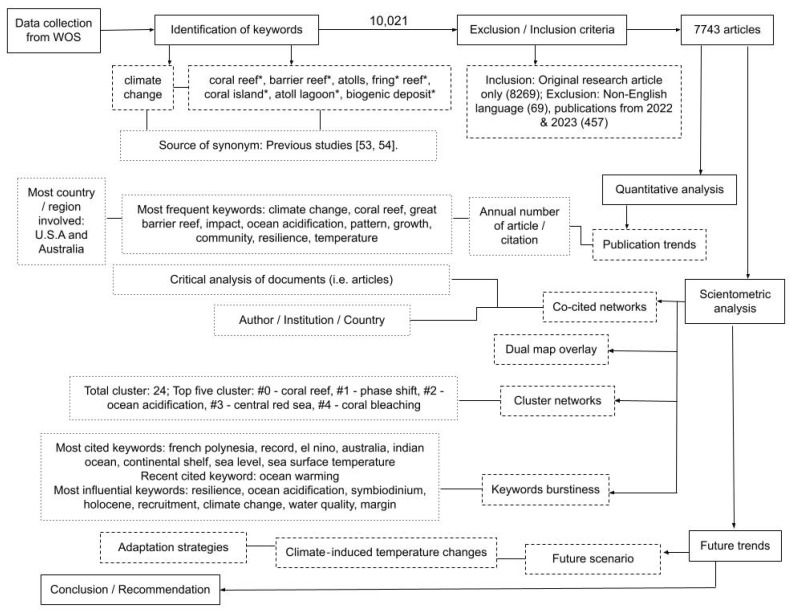
Article structure diagram.

This review addresses the question, “How have coral reefs been affected by climate change and their interactions using bibliometrics?”. Specific objectives were to assess the literature in terms of (i) the annual number of articles published, (ii) countries/regions involved in the field, (iii) research topics, (iv) cocited networks (i.e., frequency of two different documents are cited together in other documents), (v) cluster networks, (vi) research topic (i.e., keywords) burstiness, (vii) dual map overlay, and (viii) the future trends of the knowledge domain (i.e., coral reef and climate change). The main article structure diagram is shared in Figure 2.

## 4. Systematic Data Collection

This study was analyzed based on the Web of Science database Core Collection (WOSCC) on 16 November 2022. Keywords used for data collection comprised terms related to climate change [53] and coral reefs [54]. CiteSpace, 6.1.4, version 64-bit for Windows, was used to visualize current trends. The search string are as follows: CLIMATE CHANGE: (“climat* chang*”) OR (“global warm*”) OR (“seasonal* variat*”) OR (“extrem* event*”) OR (“environment* variab*”) OR (“anthropogenic effect*”) OR (“greenhouse effect*”) OR (“sea level ris*”) OR (erosio*) OR (“agricult* run-off”) OR (“weather* variab*”) OR (“weather* extrem*”) OR (“extreme* climat*”) OR (“environment* impact*”) OR (“environment* chang*”) OR (“anthropogenic stres*”) OR (“temperature ris*”) OR (“temperature effect*”) OR (“warm* ocean”) OR (“sea surface* temperat*”) OR (heatwav*) OR (acidific*) OR (hurrican*) OR (“el nino”) OR (“el-nino”) OR (“la nina”) OR (la-nina) OR (drought*) OR (flood*) OR (“high precipit*”) OR (“heavy rainfall*”) OR (“CO_2_ concentrat*”) OR (“melt* of the glacier*”) OR (“melt* ice*”) OR (“therm* stress*”) OR (“drought”) OR (“hypoxia”) AND CORAL REEF: (“coral reef*”) OR (“barrier reef*”) OR (“atolls”) OR (“fring* reef*”) OR (“coral island*”) OR (“atoll lagoon*”) OR (“biogenic deposit*”). 

## 5. Evolution of the Literature

### 5.1. Global Publication

**Figure 3:**
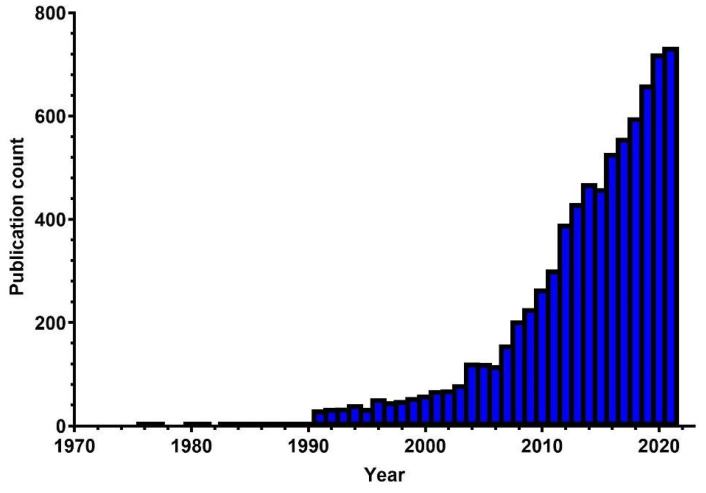
Number of original research articles on the impact of climate change on coral reefs published annually from 1977 until 2021.

**Figure 4:**
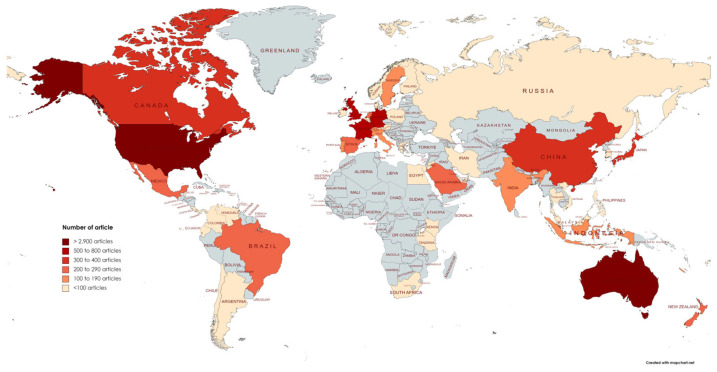
Nations publishing the most research on coral reefs and climate change generated from the freely editable map chart website (https://www.mapchart.net/world.html (accessed on 10 January 2023)).

The coral reef and climate change field showed an increase in published articles, indicating that the research is in rising momentum (Figure 3). Since 2006, studies on coral reef and climate change have been published, amounting to approximately 1.5% to almost 10% of total articles on the WoS platform in 2021, especially in the WoS Core Collection (WOSCC) database. The field entered an accelerated uptrend phase in 2016, and it is anticipated that this phase will last for the next 5 to 10 years for research publications and citations. This could be because the WOSCC added a new edition of the Emerging Sources Citation Index (ESCI) database in 2015 [55]. The leading countries in coral reef and climate change research are shown in Figure 4. The USA and Australia produced the most publications, with 2940 and 2933 articles, respectively, more than triple the output of third-ranked England, with approximately 719 articles. The USA and Australia contributed more than 75% of all publications. The study also found that there is a lack of studies conducted in the African regions. As expected, countries without coastal areas, such as Kazakhstan and Mongolia, showed no articles published in the coral reef and climate change fields. Additionally, most island nations, such as Japan, New Zealand, Australia, or Cuba, contributed to the knowledge of the coral reef and climate change.

### 5.2. Leading Institutions, Funding, and Authorship Distribution

**Figure 5:**
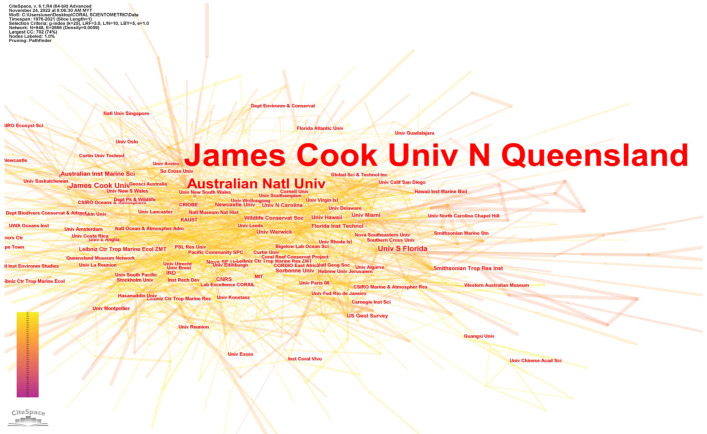
Collaborative network of institutions researching coral reefs and climate change from 1976 to 2021.

Ellegard and Wallin [46] opined that the distribution of research institutions is a useful indicator of academic support for a discipline. The network of institutions generated 948 nodes (i.e., group of entities) and 2666 collaborative links among institutions conducting research (Figure 4). The 4159 documented affiliations reflect the importance of this field in academia and the intensity of the investigations. Institutions in this collaborative network (a collaborative affiliation appeared in the article) that have contributed the most to this field are shown in Figure 5 and Table 1. An analysis of 7743 publications related to coral reefs and climate change identified a total of 29,890 affiliations. The top 20 institutions contributed to 7231 affiliations, which accounted for nearly 24% of all publications. Specifically, James Cook University was found to have contributed the highest number of publications (1119, 14% of the total), followed by the Australian Institute of Marine Science and the University of Queensland. These findings suggest that a small number of institutions have played a significant role in the research on the impact of climate change on coral reefs.

Table 2 lists the major funding agencies cited by publications in this field. The Australian Research Council funded 1001 publications, or nearly 13% of the total. Australian institutions have been at the forefront of climate change research partly because they have had greater access to funding. For instance, the Great Barrier Reef Foundation (GBRF) was awarded USD 443.3 million for the Reef Trust project between 2018 and 2019, the largest investment in reef protection to date. In 2019 and 2020, the budget allocated USD twenty-three million dollars for water quality projects, USD 4.33 million for Crown-of-Thorns (COT) control, USD 16.3 million for reef protection aimed at supporting traditional owners, USD 2.6 million for community reef protection, and USD 1.5 million for integrated monitoring and reporting. This funding allocation was based on the 2019 Great Barrier Reef Foundation’s budget plan (GBRF, 2019). In addition to these investments, the Australian Government also dedicated USD 6 million in 2018 to support the Reef Restoration and Adaptation Program’s concept feasibility phase. This program aimed to investigate the most effective science and technology options for restoring the reef, including methods for cooling and shading reef structures, coral reproduction and recruitment, biocontrol, field treatments, and coral plantation initiatives [56]. The ARC Centre of Excellence for Coral Reef Studies, located at James Cook University, conducts cutting-edge research on coral reefs. With strong collaborative ties to 24 other institutions in nine countries, it is Australia’s top contributor to coral reef sciences. Five of the ten most prominent scientists identified by CiteSpace analysis are associated with the ARC Centre of Excellence for Coral Reef Studies.

The network of authors generated in CiteSpace is a valuable tool for understanding collaborations and identifying potential future collaborations within a given field. In this study, the network of authors in the field of coral reefs and climate change generated 1680 nodes and 6931 links, where nodes represent authors with the number of publications, and links between nodes represent collaborations between authors. As a general rule, a higher number of nodes generated from CiteSpace indicates a greater degree of collaboration between authors.

The top authors in Table 3 are crucial indicators of further scientific collaboration and advancement within the field of coral reefs and climate change. However, it is important to note that while first authorship is typically assigned to the individual who made the most significant contribution to the research, this does not mean that coauthors’ contributions are any less significant or valuable. Coauthors can provide specialized expertise, contribute to data analysis and interpretation, or support the project through funding, logistical support, or other means. The authorship order and the number of authors on a publication may vary based on the specific circumstances of the research project and the agreed-upon conventions of the discipline. Ultimately, understanding the network of authors and their collaborations can help identify potential areas of future research and scientific collaboration within the coral reefs and climate change.

Emeritus Professor Terry Hughes of James Cook University, who served as the ARC Centre of Excellence for Coral Reef Studies Director from 2005 to 2020, topped the list. In 2016, Nature named Hughes one of the “10 people who mattered this year” for addressing the widespread coral bleaching event brought on by climate change. Hughes’s research has led to practical solutions to improve marine environmental management [57]. His work on the effects of climate change on coral reefs has been widely cited, especially his paper on the resistance of some coral reefs to climate change and anthropogenic factors [58]. Second on the list was Professor Ove Hoegh-Guldberg from the University of Queensland (UQ), Australia. He serves as Director of the Global Change Institute at UQ and also as a Chief Investigator at the ARC Centre of Excellence for Coral Reef Studies [59]. Dr. Katharina Fabricus is a coral reef ecologist and a Senior Principal Research Scientist at the Australian Institute of Marine Science [60]. Many of her highly cited publications are on topics related to ocean acidification [61,62,63,64], the impacts of water quality on coral reefs [65,66] and understanding the effects of terrestrial run-off on coral reefs [67,68]. 

Dr. Peter W. Glynn, from the National Center for Coral Reef Research, University of Miami, was among the pioneers in analyzing and reporting the impacts of the 1982–1983 El Niño warming event on Eastern Pacific coral reefs [69]. This was followed by Tim McClanahan, a senior conservation zoologist at the Wildlife Conservation Society and also an associate at the ARC Centre of Excellence for Coral Reef Studies [70]. His global study of more than 2500 reefs produced a Bayesian hierarchical model to predict how reef fish biomass is related to 18 socioeconomic drivers and environmental conditions [71]. Dr. Peter Mumby is a coral reef biologist from the University of Queensland and also a Chief Investigator at the ARC Centre of Excellence for Coral Reef Studies [72]. He collaborated with Professor Ove Hoegh-Guldberg to publish “Coral Reefs under Rapid Climate Change and Ocean Acidification”, which is one of the most cited papers in the field (see Table 4) [22]. Another study published in *Nature* reported the resilience of Caribbean coral reefs against moderate hurricanes [73]. Dr. David Bellwood, an Australian Laureate Fellow and Distinguished Professor at James Cook University [74], has reported on the effects of climate change on coral reef ecosystems, even though his primary research interests are in biology and the evolution of reef fish [58,75,76]. 

Dr. John Pandolfi from the University of Queensland is a paleoecologist and a Chief investigator at ARC. His research integrates long-term ecological and environmental time series data to discover past and future influences of natural variability, human impact, and climate change on coral reef resilience. Among his highly cited works is a projection of the future of coral reefs under global warming and ocean acidification [20]. Dr. Kenneth RN Anthony, an associate scientist at the Australian Institute of Marine Science and director of Environmental Strategies ES5, has published widely on ocean acidification [77,78,79]. Dr. John Bruno, from the University of North Carolina, is a marine ecologist focusing on the impacts of climate change on marine ecosystems, particularly coral reef ecology. His publication with Dr. Ove Hoegh-Guldberg, on the effects of climate change on global marine ecosystems is one of his most cited works [80].

Next on the list is Emeritus Professor Barbara E. Brown from Newcastle University, who conducted extensive research on coral bleaching, specifically on the role of zooxanthellae [81]. Next on the list is Professor Andrew C. Baker, a marine biologist at the University of Miami, who studies coral reefs and climate change. He leads the Coral Reef Futures Lab and focuses on developing and testing methods to increase coral reef resilience [82].

Glenn De’ath and Ray Berkelmans, both from The Australian Institute of Marine Science, are also highly cited for their research on coral reefs. De’ath’s work involves statistics and ecology, specifically on the Great Barrier Reef coral cover decline [83], while Berkelmans’ research focuses on thermal stress, adaptation to climate warming, the resilience of reef communities, and upwelling [84].

Joan Kleypas, a Senior Scientist from the National Center for Atmospheric Research, is also on the list, and her highly cited works revolve around the impact of ocean acidification on coral reefs [85,86]. Next is Professor Nick Graham from Lancaster University, who assesses the impacts of climate-induced coral bleaching on coral reef fish assemblages, fisheries, and ecosystem stability [87].

Emeritus Professor Michael Lesser from the University of New Hampshire is also highly cited for his work on climate change-related stressors’ biochemical and physiological impacts on coral reefs [88]. Professor Joseph Loya from Tel Aviv University quantifies changes in biodiversity and assesses reef health [89,90], while Professor Peter Edmunds focuses on the physiological ecology of tropical coral reefs [91].

Lastly, Toby Gardner is a Senior Research Fellow from Stockholm Environment Institute, known for his extensive work on Caribbean corals. He co-leads SEI’s Initiative on producer-to-consumer sustainability and the transparency for sustainable economies platform. His long-term observations revealed that the coral cover of the Caribbean basin declined by 80% in just thirty years [92].

### 5.3. Emerging Research Disciplines 

**Figure 6:**
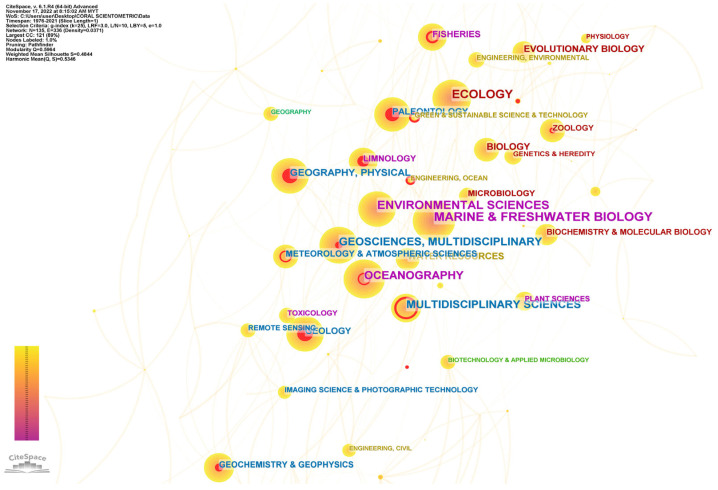
Network of linked research disciplines. The sizes of the modes are proportional to the frequency of the subject category cooccurrence. The thickness of the lines between the two nodes is proportional to the strength of the linkages between the two research disciplines.

CiteSpace’s “Category” node type was used to generate a visual map showing research disciplinary categories represented by papers addressing issues related to climate change’s impact on coral reefs. The centrality of a network (i.e., the center of collaborative activities) comprising 135 nodes and 336 links was computed after the data were simplified and merged (i.e., automatically generated from the CiteSpace algorithm and programming) (Figure 6). The five disciplines with the most publications in descending order were marine and freshwater biology, environmental sciences, ecology, oceanography, and geosciences. The study of coral reefs is a multifaceted research topic that includes many fields of study, as demonstrated by the distribution map. Disciplines in related subjects such as biodiversity conservation, geography, physical sciences, biology, evolutionary biology, geology, paleontology, and water resources, show strong connections, represented by the sizes of the nodes. The number of published papers is comparably low in some research disciplines, such as toxicology, biotechnology and applied microbiology, green and sustainable science and technology, and biochemistry and molecular biology. However, the relatively high betweenness centrality values of these fields suggest their significant contribution to interdisciplinary research, signifying their pivotal position in the scientific network. This centrality may also hint at their potential for future development and advancement in the field.

### 5.4. Research Cluster Analysis

**Figure 7:**
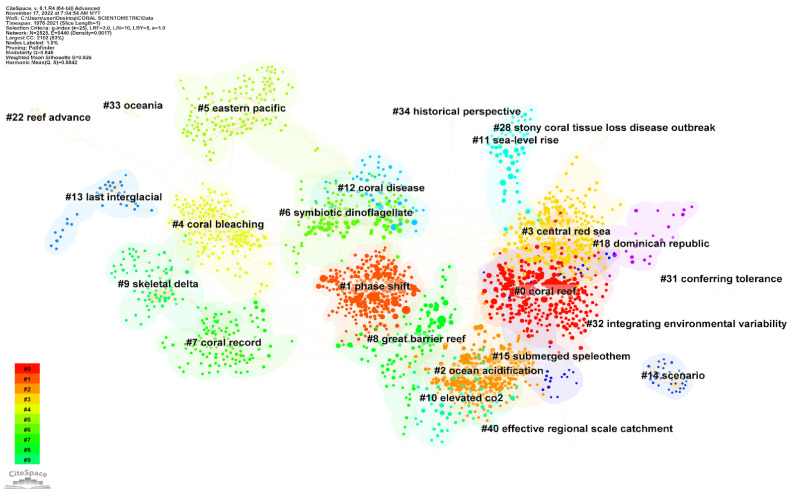
The reference co-citation research cluster network. Based on a one-year interval, a 24-cluster network of document co-citation with burst detection from 1976 to 2021. Node sizes are proportional to the frequency of the publications’ co-citations.

Cluster analysis is a popular method of statistical data analysis and knowledge discovery because of its ability to uncover latent semantic themes in textual data [93,94]. Cluster analysis can divide a large body of research data into various units based on the relative degree of term correlation, making it easier to identify the research themes, trends, and connections within a given field of study [94,95]. A cluster’s homogeneity can be quantified using an index called the mean silhouette, with values ranging from −1 to 1. The average silhouette value for each cluster was determined using CiteSpace. The higher the value, the more similar the cluster’s members are to one another [96]. The network showed 24 clusters in the context of the scientometric analysis mapping the link between climate change and coral reefs (Figure 7).

The largest cluster (#0) has 291 members (i.e., number of publications) and a silhouette value of 0.863 and is labeled as “coral reef.” The most cited article of this cluster is by Gilmour et al. [97]. They monitored and assessed the impacts of the 2016 heat stress event on Western Australian coral reefs. They found that mass bleaching in 2016 reduced coral cover by 70% at Scott Reef and caused widespread mortality (>30%) at Christmas Island, Ashmore Reef, and inshore reefs in southern Kimberley. A coral phase shift is characterized by a rapid decline in coral abundance or cover and an accompanying rise in non-reef-building organisms, like algae and soft corals [98,99]. The second largest cluster (#1) has 247 members and a silhouette value of 0.876, labeled as “phase shift.” This publication by Brodie et al. [100], entitled “Terrestrial pollutant run-off to the Great Barrier Reef: An update of issues, priorities and management responses”, is the most cited article in this cluster. They addressed findings from studies of problems caused by surface run-off of pollutants like nitrates from fertilizers, herbicides from crops, etc. Within the cluster of the study, there are three different types of management generated automatically and have mentioned (i) Reef Plant 2009, (ii) Reef Rescue, and the (iii) Reef Protection Package in the analysis. These topics are just some of the initiatives set up to continuously monitor and report on levels of discharges into the Great Barrier Reef. Multiple observations of specific facets of the topic have been published; Hughes [101]; McManus and Polsenberg [102]; Idjadi et al. [103]; Norström et al. [104]; Graham et al. [105]; Crisp et al. [106]. Many anthropogenic stressors have been linked to this phenomenon [106,107,108]. Nutrients play a pivotal role in conceptual models of how coral reef communities form. These studies show that corals have a competitive advantage over macroalgae in low nutrient conditions but that the advantage shifts to macroalgae in higher nutrient conditions [102,109]. Siltation, resulting in mud-bacterial complexes, collectively known as “marine snow,” is another factor that hinders coral growth. In addition, excess nutrients resulting in plankton blooms reduce light, thereby inhibiting coral growth [110]. 

The fourth largest cluster (#3), “ocean acidification”, has 233 members and a silhouette value of 0.959. The most cited article of this cluster is Bates [111], which reported twenty years (1996 to 2016) of marine carbon cycle observations at Devils Hole, Bermuda. Her findings shed light on the dynamic nature of biogeochemical processes like primary production, respiration, calcification, and CaCO_3_ deposition in the Bermuda reef system. During this period, neither warming nor cooling of any significance was observed. However, increases in inorganic carbon in onshore waters were primarily due to increased salinity (45%), uptake of anthropogenic CO_2_ (25%), and changes in Bermuda reef biogeochemical processes (30%). Increases in atmospheric carbon dioxide concentrations result in the absorption of more carbon dioxide by oceans, which in turn causes a decrease in pH [112,113,114,115]. The majority of research on ocean acidification has focused on the impact of changes in ocean chemistry towards suboptimal states of aragonite and calcite saturation on the calcification processes of pelagic and benthic organisms [77,116,117,118,119]. However, it is likely that ocean acidification also has an effect on other physiological processes, such as growth and reproduction in significant reef-building species [77].

The Indo-Pacific region’s Coral Triangle, the world’s epicenter of marine biodiversity [120,121], is predicted to become a “marginal” coral habitat between 2020 and 2050 unless CO_2_ emissions are reduced [122,123]. In addition to reducing coral diversity, acidification also results in a decline in shellfish and fish species due to the loss of reef structure, which provides habitat for these other species and reduces the reefs’ capacity to mitigate the effects of storm waves and erosion [122,124]. Ocean acidification has a devastating impact on the economies of ocean-dependent sectors of the global economy. Previous studies have provided estimates of the economic impact of ocean acidification on marine mollusk and shellfish production, as well as the bioeconomic costs associated with coral reef damage [125]. These studies have shed light on the detrimental effects of ocean acidification on marine ecosystems, which in turn, can have severe economic implications. Estimating these costs can aid in developing policies aimed at reducing the negative effects of ocean acidification and promoting the sustainable use of marine resources. For instance, according to a study by Narita et al. [126], the global annual loss of mollusk production due to the fact of ocean acidification could amount to between USD 6 billion and USD 100 billion. Commercially valuable finfish populations will suffer as a result of global ocean changes that reduce coral reef coverage, resulting in a loss of habitat, reduced availability of prey, and increased predation [125,127,128]. The scientometric analysis has identified four prominent clusters, also referred to as topics, which represent distinct research areas based on their geographic location. These clusters include the “central red sea” (#3), the “eastern pacific” (#5), the “great barrier reef” (#8), and the “Dominican Republic” (#18). These geographic regions are frequently cited in scientific research as they represent the study location of many relevant studies. 

The most cited article of the “Red Sea” cluster is by Osman et al. [129], which mapped coral microbiome composition along the northern Red Sea. The Red Sea is a distinctive body of water that is an evaporative basin with a high salinity above 38 ppt [130,131]. It is home to some of the world’s most thriving and productive coral reef ecosystems [132]. Osman et al. [129] research offered a fresh understanding of the coral microbiome’s exclusive and endemic characteristics along the northern Red Sea refugia. They looked into the surface mucus layer (SML) for bacterial communities from six dominant coral species and discovered five novel algal endosymbionts. Over the past four decades, the average annual sea surface temperature in most of the world’s tropics and subtropics has risen between 0.4 °C and 1 °C. However, in the central Red Sea, where reef growth and scleractinian coral diversity are abundant, warming is more extensive than the observed mean tropical temperature increase [133]. The 2010 “Thuwal bleaching” in the central Red Sea was caused by a temperature rise of 10–11 °C, the largest coral bleaching event ever recorded. Furby et al. [131] conducted a survey and found that the “Thuwal bleaching” event caused more severe bleaching of inshore reefs (74% of hard corals were bleached) than offshore reefs (14% of hard corals were bleached). One mechanism that can lead to higher tolerance is repeated exposure to thermal stress [134,135]. Based on current knowledge, it is hypothesized that the reefs in the Red Sea will be relatively resistant to bleaching as sea temperatures rise, as noted in a study by Grimsditch and Salm [136]. However, reports indicate that bleaching is beginning to occur in the Red Sea, as documented by Kleinhaus et al. [137]. For instance, Rich et al. [138] reported a winter bleaching event in the central Red Sea in January 2020 due to sea surface temperatures (SSTs) falling below 18 °C. Additionally, inshore bleaching events in the central Arabian Red Sea were observed during the “3rd global coral bleaching event” in 2015, as reported by Monroe et al. [139].

The Eastern Tropical Pacific (ETP) comprises the ocean basin extending from the Gulf of California in México to Peru and includes areas of the continental shelf and offshore islands (Coco Island, the Galápagos Islands, the Revillagigedo Archipelago and Clipperton Atoll). The most cited article of the cluster “Eastern Pacific” is Spencer [140], which discussed potentialities, uncertainties and complexities in the response of coral reefs to future sea-level rise of reef islands in the Pacific Ocean and the Caribbean Sea. Throughout the Holocene, sea levels rose without being stabilized, and reefs in the Caribbean grew in tandem with these elevation changes [141]. The once structurally complex coral reefs in the Caribbean have suffered a dramatic decline since the 1970s, with only a minority of reefs maintaining a mean live coral cover of 10% or more [142]. A strong hurricane season brought on by unusually warm waters in the tropical Atlantic, and the Caribbean in 2005 caused the worst bleaching event ever observed in the basin [143]. There was a 60% decline in coral cover on reefs in the US Virgin Islands due to the fact of a severe disease outbreak brought on by the 2005 bleaching events in the Caribbean region, as reported by Miller et al. [144].

The Great Barrier Reef is the largest coral reef ecosystem, with over 348,000 km^2^ of coverage consisting of 2900 individual reefs and 900 islands stretching over 2300 kilometers [145]. Three major coral bleaching events within a span of five years (2016, 2017, and 2020) along with the effects of severe tropical cyclones, poor water quality from catchment run-off, population growth and urbanization, overexploitation of marine resources, and habitat loss have all been the factors towards the degradation of coral reefs in the Great Barrier Reefs [56,146]. Cluster #4, which is the fifth largest cluster, contains 176 publications and has a silhouette value of 0.9. This cluster is strongly associated with cluster #6, “symbiotic dinoflagellate,” and cluster #12, “coral disease”. The high silhouette value of 1.0 indicates that there is a focused field of study in the context of coral reefs and climate change. The most cited article in cluster #4 is by Reaser et al. [147] on scientific findings and policy recommendations for coral bleaching and global climate change. Coral bleaching, which is the ability of animals with a symbiotic relationship with Symbiodinium to turn white, is an important issue associated with climate change-based literature. According to Douglas [148], all animals that have a symbiotic association with the dinoflagellate algae of the Symbiodinium genus, which are also referred to as zooxanthellae, have the ability to undergo bleaching. Symbiodinium have been reported to form extracellular symbioses with giant clams and intracellular symbioses with various organisms, including corals, anemones, jellyfish, nudibranchs, ciliophora, foraminifera, zoanthids, and sponges [149,150].

Fujise et al. [151] reported that the expulsion mechanisms of Symbiodinium were temperature-dependent; however, under non-thermal stress conditions, the expulsions of this algae were part of a regulatory mechanism to maintain a constant Symbiodinium density. In response to moderate thermal stress, Symbiodinium becomes damaged, and corals either selectively digest or expel the damaged cells. During extended periods of thermal stress, damaged Symbiodinium may accumulate in coral tissues, resulting in coral bleaching. Multiple factors have been shown to cause bleaching, including high oxidative stress [152], intense light [153], high temperature [154], low salinity [155], sedimentation [156], pollutants [157], decreased seawater temperature [158], diseases [159], bacterial infection [160,161], and ENSO-related marine heatwave events [162,163]. Degree heating weeks (DHW), defined as 1°C above the long-term climate level for the warmest month at a given locality, have become a common global predictor of bleaching [164]. Severe bleaching is typical at 8 DHW and above [165,166]. A global analysis report of coral bleaching from 1998 to 2017 [166] found that coral bleaching was most prevalent in regions with high-intensity and high-frequency thermal-stress anomalies. In areas where sea-surface temperature (SST) anomalies varied greatly, such as the Gulf of Aqaba region [167], the Caribbean Sea [168], and the Indo-Pacific [169], coral communities were significantly less susceptible to coral bleaching [166].

Globally, coral reefs have been threatened by coral disease, which is now recognized as one of the biggest threats to these ecosystems [170]. Similar to bleaching, coral disease was not considered a severe threat to coral reefs until recently [170], despite its first documentation in 1965 [171]. Since their initial descriptions, both the variety of coral diseases and the number of reported cases have skyrocketed [172,173]. Approximately 76% of all coral diseases described worldwide are found within this relatively small basin, leading experts to label the Caribbean a “hot spot” for disease [174]. For example, two dominant Acropora species in the Caribbean have been replaced by low-encrusting Agaricia due to the fact of coral disease [175,176]. Common coral diseases include Black band disease, which is caused by increased seawater temperature and anthropogenic factors, ciliates cause the Brown band disease, Cyanobacteria cause the Red band disease, and the White plague is caused by a bacterial infection [177].

### 5.5. Timeline Co-citation Analysis 

**Figure 8:**
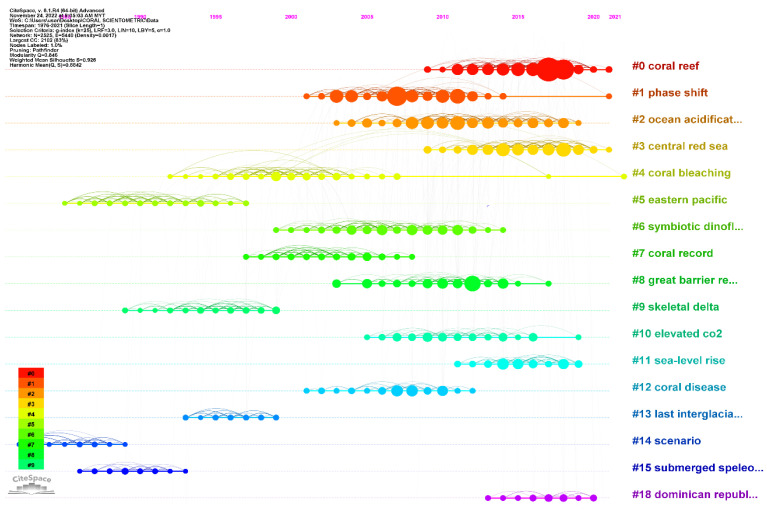
A timeline co-citation analysis. Nodes represent references, whereas lines represent connections between those references. Larger nodes indicate higher frequencies of citations. References with strong citation bursts are shown as red circles, whereas references with high centrality are shown as yellow circles. Longer line segments indicate longer time spans.

The timeline for the document co-citation analysis is an important indicator to explain the period when the study got the attention of the researcher worldwide (Figure 8). From 2010 to 2021, there have been bursts in citations for research clusters on (#0) “coral reefs”, (#2) “ocean acidification”, (#3) “central sea”, (#11) “sea level rise”, and (#5) “eastern pacific”. When taken as a whole, these studies shed light on the growing interest in studying the effects of ocean acidification and sea level rise on coral reefs, with particular attention paid to the plight of these ecosystems within the eastern pacific area, such as in the central Red Sea and the Dominican Republic.

### 5.6. Highly Cited Articles in the Field

CiteSpace’s visualized analysis of 7743 publications yielded a co-citation network (frequency of two different documents are cited together in other documents) with 2525 cited documents (nodes) and 5440 links or connections indicating co-citations between nodes [178]. The larger the node, the more often a document is cited, demonstrating its impact on coral reef and climate change research. Document co-citation analysis locates essential literature. The given references (in the article) were the most cited among 7743 Web of Science references. Table 4 presents the twenty most cited references based on co-citation analysis along with their frequency, burstiness, and centrality indices. An increase in citations reflects increased interest in that topic. “Citation bursts” demonstrate correlations between publications and sudden increases in citations. When comparing clusters, the centrality index indicates how well they are connected (i.e., coral reef and climate change). An elevated centrality score indicates that the publication is located between two or more sizable subclusters [179]. Dr. Terry Hughes’s research was widely cited, with three of his Nature and one of his Science publications ranking among the top five most-cited references. The publication entitled Global warming and recurrent mass bleaching of corals topped the list with a frequency index of 546, a burst index of 157 and a centrality index of 0.7. The burst in citation period of this publication was from 2018 to 2021. The findings were based on the third global-scale pan-tropical coral bleaching episode that occurred between 2015 and 2016. The reef ecosystem of eastern and western Australia was studied using aerial and underwater surveys along with sea surface temperatures obtained from satellites. According to their findings, the devastating bleaching event in 2016 was only slightly impacted by water quality and fishing pressure, indicating that local reef protection offers little to no protection against extreme heat. 

The second paper on the list, with a frequency index of 359 and burst index of 125, was a global study analyzing the bleaching records of 100 globally distributed reefs from 1980 to 2016 [28]. According to their findings, mass coral bleaching events happen every year regardless of the presence or absence of El Nino. They forecast that the intervals between recurrent events will eventually become too short to permit a complete recovery of mature coral assemblages, typically taking 10 to 15 years to reach the fastest-growing species. They warned that if temperatures rise by 1.5 or 2 degrees Celsius above preindustrial levels, it will exacerbate the already severe decline of coral reefs around the world. Similar findings were found in another study of his that also appeared on the list of the most-cited research. Research into the effects of climate change on coral reef ecosystems, with a special emphasis on the Great Barrier Reef, ranked fifth [28]. They found that the Great Barrier Reef’s 2016 record-breaking heatwave had caused widespread loss of functionally diverse corals across the reef’s most remote and pristine regions. Ranked third on the list was the study by Hoegh-Guldberg et al. [22], which investigated the effects of climate change and ocean acidification on coral reefs. This study was closely linked to the 3rd (#2) research cluster, also known as “ocean acidification”. The research review presented future scenarios for coral reefs, which suggested increasingly detrimental impacts on various sectors, including tourism, coastal protection, and the fisheries industry. These predictions were based on the assumption that global temperatures would rise by at least 2 °C between 2050 and 2100, coupled with atmospheric carbon dioxide concentrations exceeding 500 ppm. The findings of this study emphasize the urgent need for effective measures to mitigate climate change and ocean acidification to ensure the long-term survival and sustainability of coral reefs and the associated ecosystems. The article by LaJeunesse et al. [180] on coral endosymbionts has garnered significant attention, with a citation frequency of 134, a burst index of 68, and a burst period spanning from 2013 to 2017. This publication is associated with cluster (3), also known as “symbiotic dinoflagellate”. The article describes Symbiodinium clades and proposes that the divergent evolutionary Symbiodinium “clades” correspond to genera within the Symbiodiniaceae family. The study affirms that the long evolutionary history of the Symbiodiniaceae family is appropriately acknowledged within the suggested framework. The findings of this study provide valuable insights into the evolutionary relationships and ecological functions of these endosymbionts, highlighting the critical role they play in the health and survival of coral reefs.

The list of 11 to 20 top-cited articles on climate change on coral reefs cover a wide range of topics, including ocean acidification, declining coral cover, Symbiodinium diversity, and coral reef resilience. Several studies indicate that warming trends and bleaching stress are increasing, and coral bleaching protection mechanisms are becoming less effective, ultimately leading to significant declines in coral populations. Research on the impacts of ocean acidification and warming on marine organisms, as well as the interactions between these factors, has also shed light on the mechanisms underlying the sensitivity of coral reefs to climate change [79,181,182]. Studies on the diversity, distribution and stability of Symbiodinium [183] have provided insights into the potential for coral resilience, while research on the decline of coral cover in the Indo-Pacific region has highlighted the extent and timing of this phenomenon [183,184]. These studies demonstrate that collaborative research efforts are essential to understanding the impacts of climate change on coral reefs and developing more efficient conservation and management strategies.

### 5.7. Distribution of Keywords

**Figure 9:**
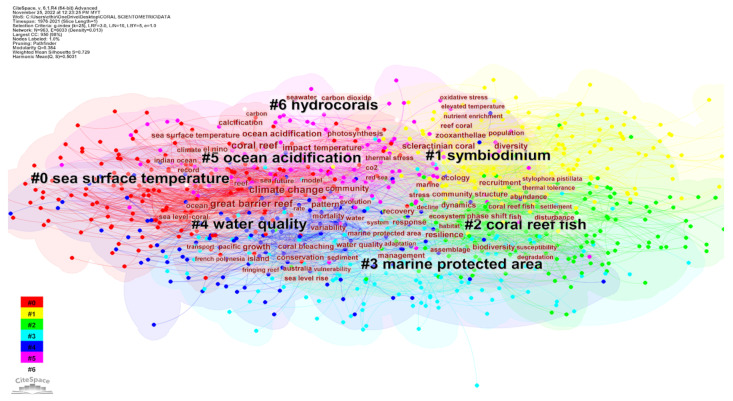
Distribution of co-cited keywords in the field of coral reef and climate change.

Using the co-cited keyword analysis performed in CiteSpace, 963 unique keywords were generated. In order to better understand the connections between these terms, a clustering tool was used to categorize them into groups (Figure 9). This generated seven major clusters consisting of “sea surface temperature”, “Symbiodinium”, “coral reef fish”, “marine protected area”, “water quality”, “ocean acidification”, and “hydrocorals”. Each of these clusters can be analyzed independently to determine which descriptors are most applicable. The major keywords used to discuss “sea surface temperature”—record, Indian ocean, and reef; “Symbiodinium”—scleractinian coral, diversity, zooxanthellae, population, nutrient enrichment, elevated pressure, and oxidative stress; “coral reef fish”—phase shift, ecosystem, disturbance, fish, dynamics, community, recruitment, abundance, thermal tolerance, *Stylophora pistillata*, and ecology; “marine protected area”—management, assemblage, biodiversity, susceptibility, degradation, adaptation, response, and recovery; “water quality”—sea level, Great barrier reef, rate, transport, coral bleaching, French Polynesia, and fringing reef; “ocean acidification”—climate, El Niño, impact temperature, coral reef, calcification, and carbon; and “hydrocorals”—seawater and carbon dioxide.

Table 5 displays the top 10 keywords with the strongest citation burst. With the exception of “ocean warming,” all the most frequently cited keywords emerged in the early 1990s and experienced a citation burst that extended until the late 2000s. The keywords identified were “French Polynesia,” “record,” “El Niño,” “Australia,” “Indian Ocean,” “Continental,” “Shelf,” “Sea level,” “Sea surface,” “temperature,” and “Island.” Notably, the keyword “ocean warming” only gained popularity in 2017, with citations peaking from 2018 to 2021. This demonstrates the significance of research on climate-related temperatures in the field of coral reefs and climate change. The keywords “Australia” and “Continental shelf” demonstrated citation bursts lasting over 15 years. In contrast, “French Polynesia” had the highest frequency of citations during a relatively shorter period, commencing in 1992 and concluding in 2006. French Polynesia, situated in the westernmost region of the South Pacific, comprises 118 islands and atolls, classified into five main clusters: the Marquesas, Society, Tuamotu, Gambier, and Austral islands [191]. These regions exhibit a north–south gradient for variables such as sea surface temperature (SST), solar insolation, evaporation, and humidity. The Millennium Coral Reef Mapping Project (MCRMP) has successfully mapped the Austral, Gambier, Society, and Tuamotu islands and atolls; however, significant research remains to be undertaken in this extensive region, which accounts for the enduring citation burst for this keyword. The findings of this study highlight the scientific interest and importance of French Polynesia as a unique and diverse region for further research and conservation efforts.

### 5.8. Dual Map Overlay

**Figure 10:**
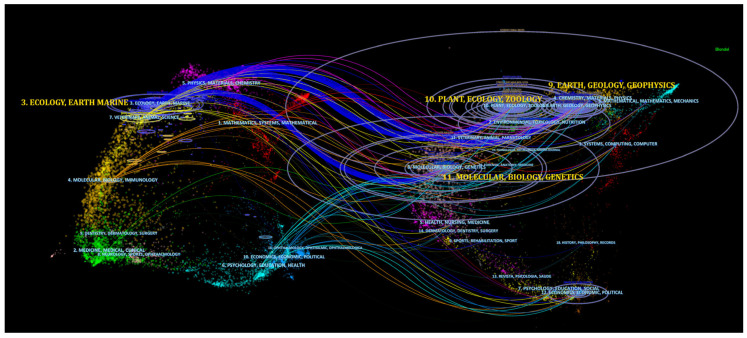
Dual-map overlay on the impact of climate change on coral reefs research.

Figure 10 illustrates the dual-map overlay of the number of articles pertaining to the type or focus of the journal. The map labels represent the research subjects covered by the journals, with the citing journals displayed on the left side and the cited journals on the right. The trajectory of the citation links provides valuable insights into inter-specialty relationships. A shift in trajectory from one region to another would indicate the influence of articles from another discipline on a specific field. In the domain of coral reef and climate change interaction, the dominant fields were found to be “ecology, earth, and marine”. The most influential discipline was “plant, ecology, and zoology”, with a z-score of 7.66, followed by “earth, geology, and geophysics”, with a z-score of 4.99 and, lastly, “molecular, biology, and genetics”, with a z-score of 2.80. These findings provide a valuable understanding of the interdisciplinary relationships within the field of coral reef and climate change research, highlighting the influence of various disciplines in shaping the current research landscape.

## 6. General Discussions

Coral reefs are a vital marine ecosystem service, providing high biodiversity and supporting the livelihoods of coastal communities. However, ocean warming and temperature are the largest threats to corals from anthropogenic climate change [192]. Between 1997 and 2018, the global average percentage of coral cover was approximately 32%, but by 2100, RCP 8.5 predicts a global decline in coral cover of 5 and 15%, equating to a relative global decline of more than 40% [115,193]. This decline is due to the fact that sea surface temperatures (SSTs) are projected to increase by more than 3 °C by the turn of the century [194]. These declines could have significant ecological and socioeconomic impacts, particularly in coastal communities that rely on coral reefs for food, tourism, and other ecosystem services.

For example, The Republic of Palau, a small Micronesian nation, has already experienced significant losses in coral reef cover [195]. Over 87% of Palau’s households are linked to coral reef-associated activities, which are critical to the country’s economic and social well-being. While tourism, particularly ecotourism, is a significant contributor to GDP growth, tax revenue, and employment, climate change-related stressors have caused a steady decline in coral reef cover. This decline has indirectly caused a major decline in tourism, threatening the country’s economic sustainability [196]. According to Barnett [197], climate change is a significant threat to food security for people in Pacific SIDS, primarily due to the decline in fisheries output resulting from the impact of climate change on total coral cover.

Apart from impacting the socioecological structure, the impact of climate change can have cascading effects on the entire reef ecosystem, affecting the abundance and diversity of other marine species that depend on corals for food and shelter. Up to 14% of species may be in imminent danger of extinction at a warming of 1.5 °C and up to 29% at a warming of 3 °C. This rise in ocean temperature will probably force coral to colonize higher latitudes that currently lack reefs [198,199,200]. However, various factors, including the need for a suitable substrate [201], connectivity to other reefs [202], ocean acidification [203], and light intensity [204], may outweigh the advantages of reefs as they expand to high latitudes [193].

Through the timeline co-citation analysis, we have observed a significant increase in research interest in the topic of climate change impacts on coral reefs between 2010 and 2022. The analysis identified several research clusters that gained traction in the scientific community, including those related to “coral reefs,” “ocean acidification,” “central red sea”, “Great Barrier Reef”, and “sea level rise”. These clusters have evolved to become research hotspots under the overarching topic of climate change impacts on coral reefs. For example, the research clusters related to the central red sea and the Great Barrier Reef have emerged as prominent research areas, given their unique characteristics and ecological importance. Similarly, the impact of ocean acidification and sea level rise on coral reefs has gained significant research interest, given their severe consequences on the health and survival of coral reefs. To better understand the impact of these research clusters, our overall discussions have been designed to incorporate the subtopics “climate change threats to coral reefs” and “adaptive strategies for coral resistance and resilience”.

### 6.1. The Threat of Climate Change to Coral Reefs: Investigating the Impacts of Temperature and Ocean Acidification

Climate-induced changes in temperature are a major threat to coral reef ecosystems, and extensive research has highlighted several key areas for investigation [53,205], with marine heatwaves, solar radiation, heat tolerance, and thermal thresholds representing the most promising areas for future research. Marine heat waves have become increasingly prevalent and intense as a result of climate change. These extreme events, characterized by prolonged periods of elevated water temperatures, significantly impact coral reef ecosystems. For instance, the mass global coral bleaching event of 2016–2017 was the most extensive and long lasting on record, as documented by Eakin et al. [206]. The event, which was associated with the El Niño Southern Oscillation (ENSO), had varying impacts on coral reefs worldwide [207], with some regions experiencing more severe bleaching than others, as reported by Kim et al. [208].

Corals are thermophilic, but their thermal tolerance is narrowly defined [169,209]. For instance, the rate of calcification increases with temperature up to a threshold level, beyond which it declines [210,211,212]. Tropical corals live close to their upper thermal limits and are, therefore, highly sensitive to periods of elevated sea surface temperatures and ocean warming [187,213]. Coral reefs in the Persian Gulf have been observed to have the highest upper-temperature thresholds of approximately 35–36 °C [214]. However, it has also been noted that these corals remain highly vulnerable to thermal stress when temperatures surpass their local maximum summer temperatures [215]. The escalating frequency and gravity of thermally induced mass bleaching events have sparked worldwide attention to the elevated temperature impacts on corals [28]. As a result, research endeavors have focused on establishing maximum thermal tolerance thresholds and variations in diverse coral species and regions and exploring potential coral refugia to brace for future ocean warming [216].

Corals rely on their symbiotic relationship with unicellular algae of the genus Symbiodinium for photosynthesis, and over 90% of their energy budget is needed for essential functions, such as calcification, tissue growth, and reproduction [212]. This critical association is threatened when corals experience thermal stress, such as elevated sea surface temperatures (SST), resulting in coral bleaching, where the algal endosymbionts are expelled. The resulting impairment and expulsion of the algal symbionts are linked to reactive oxygen species (ROS) generation from the host, the algal symbiont, or both, triggering a host immune response [217].

Protracted coral bleaching can lead to extensive coral mortality, severely affecting the ecosystem and associated reef fauna. Based on the timeline cocitation analysis, it was evident that the Red Sea (Cluster #3) and Great Barrier Reef (GBR) (cluster #8) are major research hotspots in terms of geographic regions. Although the Persian Gulf is a hot sea that supports coral reef ecosystems, the Red Sea harbors corals with greater thermal stress tolerance, with some coral genotypes capable of surviving temperatures over 5 °C above their summer maxima [216,218]. Corals in the southern end of the Red Sea are more heat resistant, surviving prolonged high temperatures, while the northern Red Sea benefits from heat-resistant genotypes that have migrated from the south [219]. The importance of broad latitudinal temperature gradients in promoting adaptation to high temperatures and exchanging heat-resistant genotypes across latitudes for genetic rescue in coral reefs is exemplified in the evolutionary history of coral reefs in the northern Red Sea [9,216]. On the other hand, the GBR, known as the world’s largest coral ecosystem, was severely impacted by the 2015–2016 climate change-amplified strong El Niño event that triggered the warmest temperatures on record. This resulted in a massive bleaching event affecting nearly 90% of reefs along the northern region, leading to a loss of approximately 30% of live coral cover in the following six months [28,220,221]. Research has increasingly linked climate change to a rise in coral diseases. Bruno et al. [222] used a high-resolution satellite dataset to investigate the relationship between temperature anomalies and coral disease on a large spatial scale of 1500 km in Australia’s Great Barrier Reef. Their findings showed a significant positive correlation between warm temperature anomalies and the incidence of the white syndrome, an emergent disease in Pacific reef-building corals. In a similar vein, Tignat-Perrier et al. [223] noted a decline in populations of two gorgonian species (*Paramuricea clavata* and *Eunicella cavolini*) found in the Mediterranean Sea due to the fact of microbial diseases during thermal stress events. These studies illustrate the growing concern that climate change is contributing to the increased incidence and severity of coral diseases, which could ultimately lead to a decline in the health of marine ecosystems.

In the past, studies on the impact of climate change on coral reefs primarily centered on the thermal tolerance of corals and the consequences of massive, abrupt coral loss on organisms associated with reefs [224]. However, research has recently shifted towards investigating the distinct and synergistic effects of ocean warming and ocean acidification resulting from increased atmospheric CO_2_ levels. The timeline co-citation analysis reveals that these emerging research fields are highly significant with recent citation bursts, as evidenced by their identification as Cluster #2 (Ocean acidification) and Cluster #10 (Elevated CO_2_), respectively.

The escalation of atmospheric carbon dioxide (CO_2_) concentrations has resulted in ocean acidification, which is among the foremost threats to coral reef ecosystems. Forecasts for 2100 anticipate a rise in CO_2_ concentrations to between 540 and 970 ppm, leading to a global decrease in seawater pH by 0.14 to 0.35 units [31,68,116,225]. As demonstrated by Fabricius et al. [68], ecological traits of coral reefs will gradually transform as seawater pH decreases to 7.8, and a decline below this level (at 750 ppm pCO_2_) would be catastrophic for these ecosystems. Ocean acidification reduces the availability of carbonate ions that corals require to form their calcium carbonate skeletons, ultimately leading to a decrease in coral calcification rates [33]. Ocean acidification has also been shown to decrease the ability of coral larvae to settle and survive [226] and increase their susceptibility to disease [227]. Research has shown that even modest increases in ocean acidity can impact the physiological processes of corals. For example, exposure to high levels of CO_2_ reduces coral growth and calcification rates [68,226]. In addition to the direct effects on coral physiology, ocean acidification can have cascading impacts on the entire coral reef ecosystem. For instance, reduced calcification by corals can reduce the complexity of the coral reef structure, potentially leading to the loss of important habitats for fish and other marine organisms [228]. Furthermore, ocean acidification can impact the symbiotic relationship between corals and their algal symbionts, potentially leading to a decline in the productivity of the reef ecosystem as a whole [229]. The combination of ocean warming and acidification is particularly concerning, as they act synergistically to exacerbate the negative impacts on coral reef ecosystems [22]. With continuing increases in atmospheric CO_2_ levels, the effects of ocean acidification on coral reefs are expected to become even more pronounced, highlighting the need for urgent action to reduce greenhouse gas emissions and protect these valuable and vulnerable ecosystems.

The rate of atmospheric CO*_2_* increase continues to accelerate, with emission scenarios predicting CO*_2_* concentrations of 540–970 ppm and a decline in seawater pH by 0.14–0.35 units globally for 2100 [68,225]. Fabricius et al. [68] demonstrated that many ecological properties in coral reefs will gradually change as pH declines to 7.8 and that it would be catastrophic for coral reefs if seawater pH dropped below 7.8 (at 750 ppm pCO_2_).

### 6.2. Adaptive Strategies for Enhancing Coral Resistance and Resilience in the Face of Climate Change

Coral resistance and resilience are scientific constructs that pertain to the capacity of coral reefs to withstand and recuperate from various stressors. Coral resistance is defined as the ability of corals to endure or tolerate perturbations and stressors, such as variations in water temperature, ocean acidification, pollution, and physical injury. Corals that possess a greater resistance to these stressors exhibit a greater ability to sustain their structure and function despite disturbances and are less prone to suffering from coral bleaching, disease, or mortality [229,230]. A myriad of studies has reported on the bleaching thresholds of corals inhabiting the Persian Gulf, despite conditions at least 2 °C higher than other coral reef ecosystems worldwide [231]. Additionally, corals from the Indo-Pacific and Caribbean regions have been found to maintain calcification rates even in low aragonite saturation states, present in naturally acidified locales [68,232]. The eastern Pacific region of Palau has revealed the thriving of reefs in waters with natural acidification, resulting from biological processes and reef system circulation patterns [232,233]. However, it is noteworthy that coral communities in Palau’s relatively acidic reef zones developed over thousands of years, fostering an inherent resistance that differs from coral communities in regions affected by higher anthropogenic interventions.

Coral resilience, in contrast, refers to the ability of coral reefs to recover from disturbances and stressors. Corals that exhibit higher resilience can reproduce, regenerate, and rebuild their structural complexity after experiencing bleaching [234]. These mechanisms are attributable to genetic diversity within coral populations and their symbiotic association with Symbiodinium algae, which are critical to their health and survival [235,236]. Genetic adaptation in corals is mediated through various factors, including the activation of heat-shock proteins, oxidoreductase enzymes, and microsporine-like amino acids. The coral surface micro-layer that absorbs UV radiation has also been identified as a significant mechanism for adaptation [180,237,238]. In-depth research on corals that thrive in the warm waters of the Persian Gulf has demonstrated their capacity for resilience, attributable to metabolic trade-offs, unique physiological characteristics, and specific genetic signatures, including a heat-specialist algal endosymbiont, *Symbiodinium thermophilum* [236,239]. *S. thermophilum* can thrive in high-temperature and high-salinity environments, allowing the coral to develop a temperature-stress-resistant phenotype [239].

Symbiodinium, a diverse group of dinoflagellates, is classified into nine clades (A–I) based on their phylogenetic characteristics [240]. Among these clades, Symbiodinium clade D has garnered attention for its exceptional thermal resilience ability, despite its relatively low representation (less than 10%) in the endosymbiotic community of coral hosts [241]. Various coral species, including fast-growing branching types, such as Acropora, Stylophora, and Pocillopora, as well as slow-growing massive, encrusting, and solitary corals, have been associated with Symbiodinium clade D [242]. The prevalence of clade D Symbiodinium in corals from the Persian Gulf has been linked to their higher thermal tolerance, particularly in comparison to corals associated with clade C, which is the dominant lineage in corals from the Great Barrier Reef and other Pacific coral reef ecosystems [243], and clade B in corals from the Atlantic [244]. These findings highlight the significance of Symbiodinium diversity in understanding the thermal resilience of coral reefs and the potential mechanisms underlying their adaptation to changing environmental conditions.

McCulloch et al. [234] explored the ability of coral species to withstand the adverse impacts of ocean acidification and global warming on coral reefs. Their study revealed that some coral species (i.e., *Stylophora pistillata* and *Porites* spp.) exhibit the capacity to increase pH levels within their calcifying fluid, crucial for the deposition of calcium carbonate and maintenance of the coral structure, even in the face of declining seawater pH levels. The study demonstrated the significance of acid-base regulation mechanisms for corals’ resilience to the effects of ocean acidification, allowing them to maintain or increase their calcification rates despite rising ocean acidification. Moreover, the study indicated that corals could acclimate to extended acidification, which enables them to maintain or increase their calcification rates by upregulating their internal pH levels, thus providing insight into potential strategies for mitigating the effects of climate change on coral reefs. A similar adaptation resilience strategy against ocean acidification was observed in cold-water scleractinian corals (i.e., *Caryophyllia smithii*, *Desmophyllum dianthus*, *Enallopsammia rostrata*, *Lophelia pertusa*, and *Madrepora oculate*) [245].

Oceanographic processes, such as upwelling and tidal currents, also play a significant role in helping corals avoid bleaching. In areas where upwelling events mix deeper, cooler water with shallow warmer water, thermal stress is reduced [246,247]; for example, in northern Galapagos during the 2015/16 ENSO [248] and Nanwan Bay, southern Taiwan, during summer [249]. Similarly, a coral reef’s ability to resist bleaching is bolstered by the elimination of potentially damaging oxygen radicals due to the swift water flow associated with tidal currents [230,250,251].

Therefore, in summary, the scientific community has identified various adaptive strategies that could enhance the resilience and resistance of coral reefs to these challenges. Going forward, it is crucial to continue ongoing research efforts to better understand the mechanisms underlying coral resilience and resistance, identify research gaps, and develop new management strategies for protecting these vital ecosystems. This can be achieved through a multidisciplinary approach that combines laboratory-based experimentation, field research, and community engagement. In addition, collaborations between the scientific community and policymakers can facilitate the implementation of evidence-based management practices that promote the resilience and resistance of coral reefs to climate change and other stressors.

## 7. Conclusions

The scientometric analysis that is presented in this article demonstrates that research on coral reefs in relation to climate change has emerged as one of the potential fields, with interest in this topic has grown steadily since the 2000s. The increasing global temperatures are posing a significant threat to coral reefs, leading to widespread coral bleaching and mortality. Moreover, changes in ocean chemistry brought on by an increase in carbon dioxide levels lead to ocean acidification, which can worsen the effects of rising temperatures on corals [22]. In addition, sea level rise and coastal development are transforming the physical structure of coral reef ecosystems, exacerbating the negative effects of the other stressors [252]. These changes are harmful not only to the coral reefs but also to the plethora of species that rely on them for survival and the communities that rely on them for livelihoods and for protecting the coast. Future challenges for developing countries like those within the coral reef triangle initiative (i.e., Indonesia, Malaysia, the Philippines, Papua New Guinea, Timor Leste, and the Solomon Islands) will center on access to funding for conservation-restoration efforts and continued monitoring studies. There are several ways that ongoing research and coordinated action can help coral reefs cope with the effects of climate change:

- Monitoring: Regular coral reef monitoring can reveal vital details about the well-being and state of the reefs as well as the effects of climate change. These data can be used to pinpoint especially vulnerable regions and monitor long-term changes. Scientists and environmentalists can detect early warning signs of coral bleaching and other detrimental effects by monitoring coral reefs, which enables them to take action before it is too late;
- Research: Collaborative research efforts can contribute to a better understanding of the impacts of climate change on coral reefs and the mechanisms underlying these impacts and can also aid in developing and rigorously testing intervention and restoration techniques for coral reefs. Given that the preservation of coral reef ecosystems requires a range of interventions, including biological, ecological, and social strategies for mitigation and adaptation [19], such research can help create more efficient restoration, conservation and management strategies;
- Conservation and management: Collaborative conservation and management efforts can assist in mitigating the effects of climate change on coral reefs. Protected areas and marine reserves, for instance, can aid in mitigating the effects of overfishing and pollution, thereby making coral reefs more resilient to the effects of climate change;
- Mitigation: joint efforts can also aid in lowering atmospheric greenhouse gas concentrations, which are primarily responsible for climate change, for instance, by collaborating with regional organizations and authorities to advance sustainable development and lower carbon emissions;
- Public education and awareness: Raising public understanding of the effects of climate change on coral reefs can encourage support for management and conservation initiatives.

## Figures and Tables

**Table 1:** Top 20 institutions in the field of coral reef and climate change.

Institution	Record Count
James Cook University (Australia)	1119
Australian Institute of Marine Science (Australia)	727
University of Queensland (Australia)	643
University of California System (USA)	449
Centre National de la Recherche Scientifique, CNRS (France)	443
UDICE French Research Universities (France)	400
National Oceanic Atmospheric Admin NOAA (USA)	379
University of Hawaii System (USA)	345
University of Western Australia (Australia)	335
Commonwealth Scientific Industrial Research Organization CSIRO (Australia)	312
Institut de Recherche pour le Développement (France)	279
State University System of Florida (USA)	270
University of Miami (USA)	242
United States Department of the Interior (USA)	199
University of Hawaii Manoa (USA)	191
King Abdullah University of Science Technology (Saudi Arabia)	185
Smithsonian Institution (USA)	182
United States Geological Survey (USA)	178
University of California San Diego (USA)	177
Australian National University (Australia)	176

**Table 2:** Top 20 funding agencies related to coral reef and climate-change-related studies.

Funding Agency	Record Count
Australian Research Council (Australia)	1001
National Science Foundation (USA)	807
Australian Government (Australia)	307
National Oceanic and Atmospheric Administration (USA)	258
UK Research Innovation (United Kingdom)	248
Natural Environment Research Council (United Kingdom)	231
National Natural Science Foundation (China)	201
European Commission of the European Union (EU)	187
National Science Foundation (NSF) Directorate for Geosciences (USA)	177
Ministry of Education Culture, Sports Science, and Technology (Japan)	161
Japan Society for The Promotion of Science (Japan)	142
Australian Institute of Marine Science (Australia)	134
CGIAR (United Nations)	126
German Research Foundation (Germany)	124
Consejo Nacional de Ciencia y Tecnología (Mexico)	117
National Council for Scientific and Technological Development (Brazil)	114
Natural Sciences and Engineering Research Council of Canada (Canada)	102
King Abdullah University of Science Technology (Saudi Arabia)	92
Agence Nationale de la Recherche (France)	84
Grants-in-Aid for Scientific Research (Kakenhi) (Japan)	78

**Table 3:** Top 20 authors in the field of coral reef and climate change.

Institution	Affiliation	Citation Count
Terry Hughes	James Cook University ARC Centre of Excellence for Coral Reef Studies	2605
Ove Hoegh-Guldberg	The University of Queensland ARC Centre of Excellence for Coral Reef Studies	2441
Katharina Fabricius	Australian Institute of Marine Science	1108
Peter W. Glynn	University of Miami	979
Tim McClanahan	The Wildlife Conservation Society ARC Centre of Excellence for Coral Reef Studies	960
Peter Mumby	The University of Queensland ARC Centre of Excellence for Coral Reef Studies	907
David R. Bellwood	James Cook University	891
John Pandolfi	The University of Queensland ARC Centre of Excellence for Coral Reef Studies	866
Kenneth R.N. Anthony	Australian Institute of Marine Science	841
John Bruno	The University of North Carolina	781
Barbara E. Brown	Newcastle University	773
Andrew C. Baker	University of Miami	749
Glenn De’ath	The Australian Institute of Marine Science	746
Joan Kleypas	National Center for Atmospheric Research	729
Ray Berkelmans	Australian Institute of Marine Science	705
Nick Graham	Lancaster University	687
Michael Lesser	The University of New Hampshire	661
Joseph Loya	Tel Aviv University	636
Peter J. Edmunds	California State University	596
Toby Gardner	Stockholm Environment Institute	570

**Table 4:** The most highly cited references about coral reefs and climate change.

Reference	Citations	Burst Index *	Burst Period	Journal	Centrality **	Cluster
Hughes et al. [185]	546	157.67	2018–2021	Nature	0.7	#0, #4
Hughes et al. [28]	359	125.52	2018–2021	Science	0	#4
Hoegh-Guldberg et al. [22]	351	166.14	2008–2012	Science	0.3	#0, #2
Hughes et al. [185]	252	87.69	2018–2021	Nature	0.4	#0, #7
Hughes et al. [23,28]	246	94.42	2019–2021	Nature	0.01	#8
De’Ath et al. [83]	204	79.65	2013–2017	PNAS	0.6	#7, #8
Pandolfi et al. [20]	175	68.19	2012–2016	Science	0.4	#0, #2
Fabricius et al. [68]	162	63.09	2012–2016	Nature Climate Change	0.05	#2
LaJeunesse et al. [180]	134	68.39	2013–2017	Current Biology	0	#6
Hughes et al. [186]	122	48.71	2011–2015	Trends in Ecology & Evolution	0.01	#0
Heron et al. [187]	118	33.85	2018–2021	Scientific Reports	0.6	#4
Ainsworth et al. [188]	117	28.75	2017–2021	Science	0.4	#0, #4
Palumbi et al. [189]	116	39.87	2015–2019	Science	0.05	#0
Baker et al. [183]	108	50.7	2010–2013	CETP	0.04	#5, #6
Anthony et al. [79]	105	45.6	2009–2013	Global Change Biology	0.4	#10
Kroeker et al. [182]	104	39.22	2014–2018	Global Change Biology	0.4	#2, #10
Zeebe and Wolf-Gladrow [181]	97	39.97	2010–2014	Gulf Professional Publishing	0.6	#4, #10
Jackson et al. [190]	96	34.08	2016–2019	Global Coral Reef Monitoring Network	0.4	#7, #18
Bruno and Selig [184]	95	44.45	2008–2012	PLoS one	0.05	#0, #7

* Burst index: the value generated from the CiteSpace indicates the level of importance of each article in the field. ** Centrality: the main focused article between cited references in the publications.

**Table 5:** Top 10 keywords with the strongest citation bursts.

Keyword	Year	Strength	Begin	End	1976–2021
French Polynesia	1992	24.51	1992	2006	▂▂▂▂▂▂▂▂▂▂▂▂▂▂▂▂▃▃▃▃▃▃▃▃▃▃▃▃▃▃▃▂▂▂▂▂▂▂▂▂▂▂▂▂▂▂
Record	1992	24.36	1992	2005	▂▂▂▂▂▂▂▂▂▂▂▂▂▂▂▂▃▃▃▃▃▃▃▃▃▃▃▃▃▃▂▂▂▂▂▂▂▂▂▂▂▂▂▂▂▂
El Niño	1995	22.08	1995	2006	▂▂▂▂▂▂▂▂▂▂▂▂▂▂▂▂▂▂▂▃▃▃▃▃▃▃▃▃▃▃▃▂▂▂▂▂▂▂▂▂▂▂▂▂▂▂
Australia	1992	21.33	1992	2007	▂▂▂▂▂▂▂▂▂▂▂▂▂▂▂▂▃▃▃▃▃▃▃▃▃▃▃▃▃▃▃▃▂▂▂▂▂▂▂▂▂▂▂▂▂▂
Indian Ocean	1990	17.72	1998	2010	▂▂▂▂▂▂▂▂▂▂▂▂▂▂▂▂▂▂▂▂▂▂▃▃▃▃▃▃▃▃▃▃▃▃▃▂▂▂▂▂▂▂▂▂▂▂
Continental shelf	1993	17.14	1993	2010	▂▂▂▂▂▂▂▂▂▂▂▂▂▂▂▂▂▃▃▃▃▃▃▃▃▃▃▃▃▃▃▃▃▃▃▂▂▂▂▂▂▂▂▂▂▂
Sea level	1991	16.43	1991	2005	▂▂▂▂▂▂▂▂▂▂▂▂▂▂▂▃▃▃▃▃▃▃▃▃▃▃▃▃▃▃▂▂▂▂▂▂▂▂▂▂▂▂▂▂▂▂
Sea surface temperature	1996	15.91	1996	2006	▂▂▂▂▂▂▂▂▂▂▂▂▂▂▂▂▂▂▂▂▃▃▃▃▃▃▃▃▃▃▃▂▂▂▂▂▂▂▂▂▂▂▂▂▂▂
Ocean warming	2017	15.09	2018	2021	▂▂▂▂▂▂▂▂▂▂▂▂▂▂▂▂▂▂▂▂▂▂▂▂▂▂▂▂▂▂▂▂▂▂▂▂▂▂▂▂▂▂▃▃▃▃
Island	1991	14.65	1991	2005	▂▂▂▂▂▂▂▂▂▂▂▂▂▂▂▃▃▃▃▃▃▃▃▃▃▃▃▃▃▃▂▂▂▂▂▂▂▂▂▂▂▂▂▂▂▂

## Data Availability

No data was generated from the study.
